# Urinary steroid profiling in women hints at a diagnostic signature of the polycystic ovary syndrome: A pilot study considering neglected steroid metabolites

**DOI:** 10.1371/journal.pone.0203903

**Published:** 2018-10-11

**Authors:** Nasser A. Dhayat, Nesa Marti, Zahraa Kollmann, Amineh Troendle, Lia Bally, Geneviève Escher, Michael Grössl, Daniel Ackermann, Belen Ponte, Menno Pruijm, Michael Müller, Bruno Vogt, Martin H. Birkhäuser, Murielle Bochud, Christa E. Flück, members of the SKIPOGH Study Group

**Affiliations:** 1 Department of Nephrology and Hypertension and Department of BioMedical Research, Inselspital, University Hospital, University of Bern, Bern, Switzerland; 2 Pediatric Endocrinology and Diabetology, Department of Pediatrics and Department of BioMedical Research, Inselspital, Bern University Hospital, University of Bern, Bern, Switzerland; 3 Department of Obstetrics and Gynecology, Inselspital, Bern University Hospital, University of Bern, Bern, Switzerland; 4 Lindenhofspital, Bern, Switzerland; 5 Department of Diabetes, Endocrinology, Clinical Nutrition and Metabolism, Inselspital, Bern University Hospital, University of Bern, Bern, Switzerland; 6 Nephrology Service, Department of Specialties of Internal Medicine, University Hospital of Geneva, Genève, Switzerland; 7 Nephrology Service, University Hospital of Lausanne, Lausanne, Switzerland; 8 Institute of Social and Preventive Medicine, University Hospital of Lausanne, Lausanne, Switzerland; Universite Clermont Auvergne, FRANCE

## Abstract

**Background:**

Although the polycystic ovary syndrome (PCOS) is the most common endocrine disorder in women with vast metabolic consequences, its etiology remains unknown and its diagnosis is still made by exclusion. This study aimed at characterizing a large number of urinary steroid hormone metabolites and enzyme activities in women with and without PCOS in order to test their value for diagnosing PCOS.

**Methods:**

Comparative steroid profiling of 24h urine collections using an established in-house gas-chromatography mass spectrometry method. Data were collected mostly prospectively. Patients were recruited in university hospitals in Switzerland. Participants were 41 women diagnosed with PCOS according to the current criteria of the Androgen Excess and PCOS Society Task Force and 66 healthy controls. Steroid profiles of women with PCOS were compared to healthy controls for absolute metabolite excretion and for substrate to product conversion ratios. The AUC for over 1.5 million combinations of metabolites was calculated in order to maximize the diagnostic accuracy in patients with PCOS. Sensitivity, specificity, PPV, and NPV were indicated for the best combinations containing 2, 3 or 4 steroid metabolites.

**Results:**

The best single discriminating steroid was androstanediol. The best combination to diagnose PCOS contained four of the forty measured metabolites, namely androstanediol, estriol, cortisol and 20βDHcortisone with AUC 0.961 (95% CI 0.926 to 0.995), sensitivity 90.2% (95% CI 76.9 to 97.3), specificity 90.8% (95% CI 81.0 to 96.5), PPV 86.0% (95% CI 72.1 to 94.7), and NPV 93.7% (95% CI 84.5 to 98.2).

**Conclusion:**

PCOS shows a specific 24h urinary steroid profile, if neglected metabolites are included in the analysis and non-conventional data analysis applied. PCOS does not share a profile with hyperandrogenic forms of congenital adrenal hyperplasias due to single steroid enzyme deficiencies. Thus PCOS diagnosis by exclusion may no longer be warranted. Whether these findings also apply to spot urine and serum, remains to be tested as a next step towards routine clinical applicability.

## Introduction

Polycystic ovary syndrome (PCOS) affects about 10% of women and may have major reproductive and metabolic consequences [1,2,3]. PCOS is diagnosed by exclusion mainly because of lack of knowledge of its complex pathomechanism. Current criteria for diagnosing PCOS by the Androgen Excess and PCOS Society comprise one, androgen excess by clinical and/or biochemical means, and two, ovulatory dysfunction and/or polycystic ovaries by morphology, under the exclusion of other etiologies [1,2]. However, PCOS diagnosis is often delayed and this affects patients’ well-being negatively [4]. PCOS patients are often insulin resistant and obese, have often a positive family history, encountered often premature adrenarche, or were born small for gestational age. Overall, hyperandrogenism seems to play an essential role in PCOS manifesting clinically as acne, hirsutism, and menstrual disturbances. Biochemically, elevated serum androgens and increased AMH and LH levels may be found, but to date there is no reliable diagnostic laboratory test for diagnosing PCOS [1,2]. Unspecific disturbances of the steroid profile are often observed, but no diagnostic pattern has been identified so far.

Androgens are produced primarily in the gonads and the adrenal cortex. In women about 25% of circulating androgens originate from the adrenals, 25% from the ovaries, and 50% from peripheral conversion of precursor steroids [5,6]. Normally, plasma testosterone concentrations in a 30 year old female are about 10-fold lower compared to an age-matched male, but may be markedly elevated with PCOS.

The classic androgen biosynthesis pathway in the adrenal cortex zona reticularis (ZR) and the human ovary is long known and follows the Δ5-pathway from cholesterol to 17-hydroxypregnenolone to dehydroepiandrosterone (DHEA), the first androgen precursor [7]. In the ZR, the theca cell of the ovary, and in peripheral tissues, DHEA is converted to androstenedione, which is thereafter mainly converted to estrogens and only in little quantities to testosterone (T), either in the ovary or in peripheral tissues. Finally, some T may be further converted to dihydrotestosterone (DHT), the most potent androgen. Recently, an alternative pathway for androgen biosynthesis has been described first in the tammar wallaby [8], then in humans [9]. In this alternative, backdoor pathway 17-hydroxyprogesterone or 17-hydroxypregnenolone is driven away from the classic pathway by 5α-3α reducing reactions yielding 17-hydroxy-allopregnanolone, which is then converted to androsterone and androstanediol or androstanedione before yielding DHT. A role for this alternative pathway has been established for the human testis [10] and the adrenal cortex [11,12]; and it has been suggested for the human ovary from immunohistochemical studies [13], and from steroid profiling in PCOS [14]. However, whether this pathway plays a role for excess androgen production awaits further confirmation. It has been reported that in PCOS increased 5α reductase activity converts androstenedione to androstanedione, which is then converted to DHT [15]. In line with that, we found increased 5α reductase expression in PCOS ovaries [13]. Furthermore, newer studies show that the adrenal ZR (and maybe the theca cells) produce 11-OH-androstenedione, which can be converted to potent androgens such as 11-ketotestosterone [16]. Accordingly, elevated serum concentrations of 11-oxygenated androgens were measured in women with PCOS [17]. But albeit all these novel findings, there is still no diagnostic laboratory test for PCOS.

Therefore in this study, we performed comprehensive steroid metabolic profiling of urine specimens obtained from PCOS women and compared it to healthy, matched controls in order to find PCOS characteristic changes for diagnostic use. We assessed 40 steroid metabolites and analyzed them for significant differences between groups looking at the level of single metabolites and at ratios characterizing enzyme activities. We also searched for androgens produced by alternative pathways. In addition, unbiased data analysis was performed by calculating systematically combinations of steroid metabolites aiming at finding a diagnostic classifier that would be able to discriminate PCOS from controls.

## Materials and methods

### Study design and participants

The study was approved by the ethics commission of the Kanton Bern, Switzerland (study ID004/07). Participants provided written informed consent. The study was partially retrospective for the PCOS group and fully prospective for the healthy control group. Study inclusion was possible for patients with a PCOS diagnosis according to the Androgen Excess and PCOS Society [1]. Females were postmenarchal (13 to 46 years), without hormonal treatments and without other disease conditions. A 24h-urine sample collection was mandatory. The matched control group was recruited in parallel with the Swiss Kidney Project on Genes in Hypertension (SKIPOGH) study [18,19], means healthy controls participated in both studies and did not have PCOS. Of the 1128 healthy SKIPOGH participants, 591 are women, 264 were ≤ 46 years at the time of urine sampling. Out of these 264 women, 187 were excluded for medication intake (e.g. anticonception), 7 for irregular periods, 3 for missing urine steroid measurements, and one for diagnosis of PCOS; leaving 66 eligible control participants.

### Sample collection and biochemical measurements

Study participants were instructed to collect 24-hour urine. Samples were stored at ≥-20°C before assessing the steroid profile with an *in-house* method of gas chromatography, mass spectrometry (GC-MS) [20]. In brief, the method comprises a pre-extraction on a Sep-Pak C18 column, an enzymatic hydrolysis following extraction on a Sep-Pak C18 cartridge, derivatization and purification on a Lipidex 5000 column. A gas chromatograph 7890A from Agilent Technologies (La Jolla, California, USA) coupled to a mass selective detector Hewlett-Packard 5975C providing selected ion monitoring (SIM) was used. Further details about the steroid compounds and the GC-MS method are reported in [20]. Fasting blood samples were analysed by standard laboratory methods. The homeostasis model assessment insulin resistance (HOMA-IR) and beta-cell function (HOMA-β) were used to assess insulin resistance and beta-cell function [21].

### Statistical analyses

All statistical analyses were performed using R (version 3.2.5; R Foundation for Statistical Computing, Vienna). All tests were two-sided and a p value <0.05 was considered statistically significant unless otherwise stated. The shape of the distribution of quantitative urinary steroid hormone metabolites and of steroid hormone ratios was visualized and transformations were applied to dependent variables in uni- and multivariable linear regression analyses. Regression models were graphically validated and revealed no obvious deviations from homoscedasticity or normality. The accuracy of different classifier to discriminate women into PCOS and healthy was assessed by the area under the curve (AUC) and its 95% CI of a receiver operating characteristic (ROC) analysis using the statistical R packages “pROC”, “ROCR”, and “Epi”. Performance of all 40 steroid metabolites and their ratios including sums and products of log, square root and square were analysed to find the best classifiers. Combinations of 2 or 3 steroid metabolites were analysed, thereby investigating far more than one million possible combinations. To increase the AUC under the ROC curve, the best discriminating combinations of 3 classifiers were further optimized by stepwise adding and omitting additional metabolites. Sensitivity-specificity versus classifier plot were created for the best classifiers to indicate the threshold where sensitivity and specificity are simultaneously maximized using the R package “OptimalCutpoints”, and the corresponding contingency tables with test characteristics were produced. Multivariable regression models containing four classifiers were described and visualized using the R package “visreg”.

## Results

### Baseline characteristics of the study population

Baseline characteristics are listed in S1 Table. The PCOS group was younger compared to controls with a median age of 27 versus 34 years (range 13–46 versus 18–46 years, *p*<0.001). BMI was not significantly different. Resting systolic blood pressure was higher in the PCOS group with a median of 115 versus 109 mmHg (range 100–140 versus 86–148 mmHg, *p*<0.01). No difference was observed for resting diastolic blood pressure. Fasting plasma glucose was similar in both groups, but serum insulin was higher in PCOS subjects with a median of 16.6 versus 3.2 mU/l (range 5.2–26.7 versus 1–19 mmHg, *p*<0.001). Accordingly, both HOMA-IR and HOMA-β were higher in the PCOS group compared to controls indicating insulin resistance in PCOS.

### 24-hour urine steroid metabolite excretion

**Fig 1:**
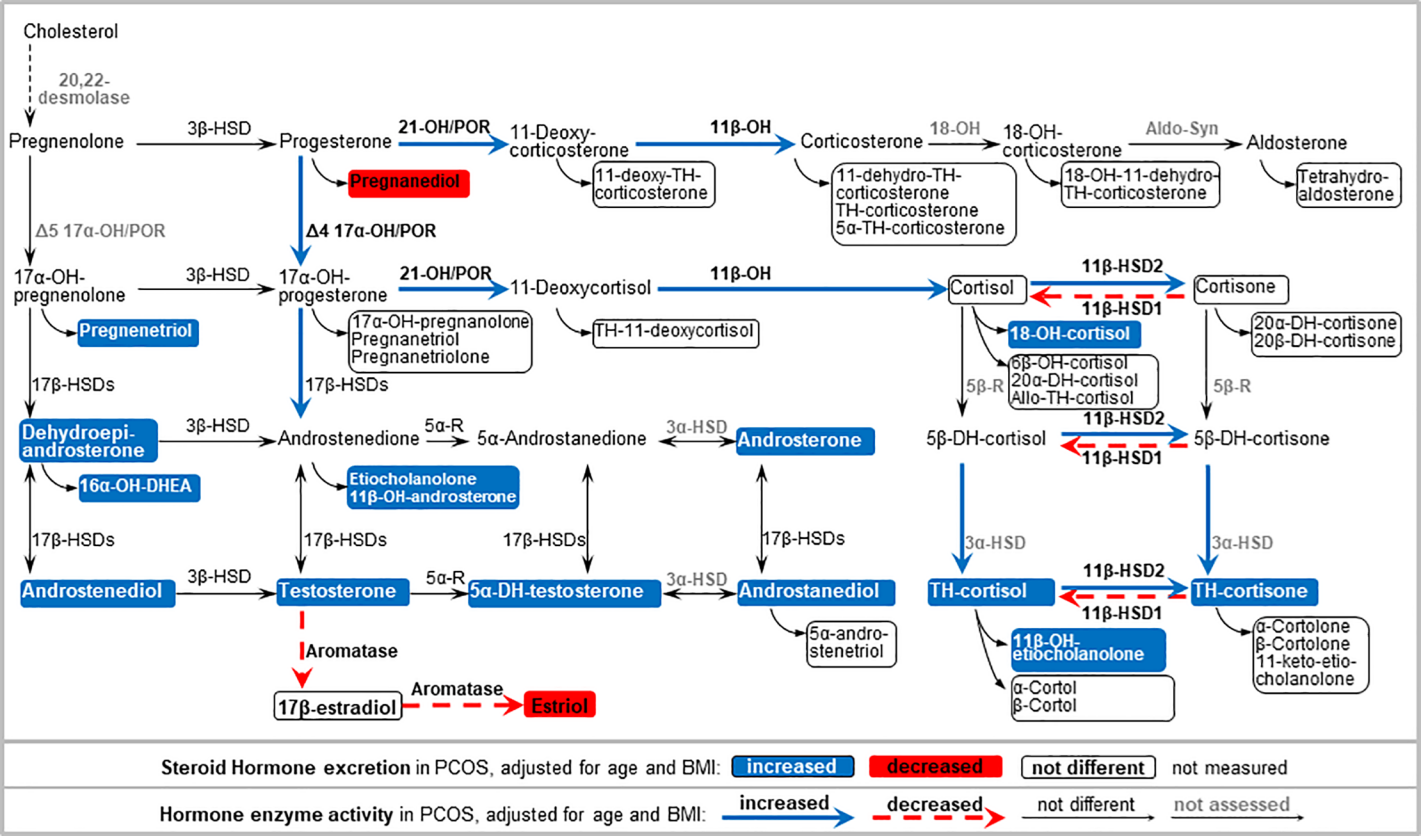
Scheme of alterations in 24-hour urine steroid excretion and steroid enzyme activities in PCOS compared to controls adjusted for age and BMI in multivariable analyses. Abbreviations: DHEA: dehydroepiandrosterone. An “OH” in enzyme names indicates a hydroxylase. An “OH” in steroid names indicates a hydroxyl group. DH: dehydro; TH: tetrahydro; HSD: hydroxysteroid dehydrogenase; POR: P450 oxidoreductase; Cyt b5: Cytochrome b5; 5α-R: 5α reductase; 5β-R: 5β reductase.

Comparison of 24-hour urine steroid metabolite excretion between PCOS and controls by Mann-Whitney U test, and by uni- and multivariable linear regression analyses is summarized in Table 1 (and S2 Table). The largest increase in median steroid metabolite excretion was found in the PCOS group for dehydroepiandrosterone (4.9-fold, *p*<0.001), androstenediol (3.0-fold, *p*<0.001), pregnenetriol (2.8-fold, *p*<0.001), 16α-OH-dehydroepiandrosterone (2.3-fold, *p*<0.001) and androstanediol (2.3-fold, *p*<0.001). Higher excretion was found in controls for pregnanediol (1.6-fold, *p* = 0.0019) and estriol (1.4-fold, *p* = 0.027). In multivariable analyses a higher excretion was found in PCOS for 14 steroid compounds, including 9 androgens and 4 glucocorticoids. Lower excretion of pregnanediol and estriol in PCOS persisted even after adjustement for age and BMI (Table 1). Results of the multivariable analyses are depicted in Fig 1.

**Table 1:** Steroid hormone excretion in women with the polycystic ovary syndrome (PCOS) compared to healthy control women. The available number of participants (N) and the distribution described by median and 25^th^-75^th^ quantile for the PCOS and control group are indicated for each steroid. Between-group differences are determined by Mann–Whitney U test (MWU) and the corresponding *P* values are indicated. Univariable and multivariable models were calculated by linear regression. Univariable models contain the PCOS-/control-group as predictor variable (with controls as reference group). Multivariable models contain in addition the covariables age and BMI. The dependent variables in the models were transformed as indicated. The β coefficients and the corresponding 95% confidence intervals (CI) are reported in the transformed scale and the corresponding *P* values are indicated. Note, only steroid hormones with a significant difference in the amount excreted in 24 hours in women with PCOS compared to healthy controls are shown here. Results for all 40 steroid hormones measured are displayed in S2 Table.

Steroid hormone, nmol/24h Dependent variable in Models	Controls			PCOS			MWU	Univariable Models			Multivariable Models		
	N	Median	25^th^-75^th^	N	Median	25^th^-75^th^	*P*	β	95% CI	*P*	β	95% CI	*P*
**Androgens and metabolites**													
dehydroepiandrosterone^a^	66	293	136–853	41	1435	390–3895	<0.001	1.27	0.712;1.83	<0.001	1.03	0.437;1.62	<0.001
16α-OH-dehydroepiandrosterone^a^	66	676	314–1213	41	1577	701–3321	<0.001	0.740	0.289;1.19	0.0015	0.740	0.289;1.19	0.0015
androstenediol^a^	66	205	125–430	41	622	405–1314	<0.001	1.07	0.705;1.43	<0.001	0.858	0.483;1.23	<0.001
testosterone^a^	63	34	21–58	33	52	34–84	0.013	0.449	0.106;0.793	0.011	0.427	0.05;0.804	0.027
5α-DH-testosterone^a^	65	36	23–55	33	56	44–88	0.0057	0.477	0.148;0.805	0.0049	0.387	0.029;0.746	0.035
androstanediol^a^	65	108	65–142	41	250	185–350	<0.001	0.930	0.735;1.13	<0.001	0.886	0.68;1.09	<0.001
androsterone^b^	57	3983	2651–5433	41	8354	4909–11808	<0.001	24.9	15.7;34	<0.001	14.7	6.31;23	<0.001
11β-OH-androsterone^b^	66	1385	1049–2048	41	2210	1618–3263	<0.001	9.80	5.35;14.2	<0.001	8.73	4.2;13.3	<0.001
etiocholanolone^b^	61	4075	2823–5709	41	5893	4558–8210	<0.001	13.5	6.3;20.6	<0.001	9.65	2.13;17.2	0.012
**Estrogens**													
estriol^a^	66	29	16–49	41	21	8–34	0.027	-0.444	-0.809;-0.079	0.018	-0.491	-0.877;-0.105	0.013
**Glucocorticoids and metabolites**													
6β-OH-cortisol^a^	66	222	147–348	41	319	189–445	0.025	0.238	-0.012;0.489	0.062	0.256	-0.016;0.529	0.065
18-OH-cortisol^b^	61	434	301–607	39	676	448–924	<0.001	5.75	3.1;8.39	<0.001	5.89	3.02;8.76	<0.001
TH-cortisol^b^	59	2770	1926–3439	41	3613	2603–4404	0.0017	8.06	2.85;13.3	0.0028	7.91	2.72;13.1	0.0032
11β-OH-etiocholanolone^b^	66	872	410–1196	40	1037	255–1640	0.51	1.87	-2.92;6.67	0.44	5.75	0.837;10.7	0.022
TH-cortisone^b^	64	5551	3394–7209	41	8559	5651–13063	<0.001	23.8	14.8;32.9	<0.001	21.2	12;30.4	<0.001

^a^Dependent variable natural log transformed in regression models.

^b^Dependent variable square root transformed in regression models.

### Steroid enzyme activities

Steroid enzyme activities were assessed by metabolite ratios as published for diagnosing various forms of congenital adrenal hyperplasias [22]. Ratios were compared between PCOS and controls by Mann–Whitney U test and by uni- and multivariable regression models (Table 2 and S3 Table). Steroid metabolite substrate to product conversion ratios representing 21-hydroxylase activity were lower in PCOS indicating an increased 21-hydroxylase activity in PCOS compared to controls. This association persisted in multivariable regression analyses adjusted for age and BMI. Thus 21-hydroxylase deficiency could be clearly excluded in our PCOS patients. 3β-hydroxysteroid dehydrogenase (3β-HSD) activity seemed lower in PCOS, but this association to PCOS disappeared after multivariable adjustment. By contrast, a higher enzyme activity was found in PCOS for the activities of 11β-hydroxylase, and the Δ4-pathway activity of 17α-hydroxylase and 17,20-lyase, as well as for the P450 oxidoreductase activity. For the activity of 17β-hydroxysteroid dehydrogenase multivariable analyses indicated no difference. Similarly, no clear difference was found for the ratio yielding androgen synthesis through the backdoor pathway or for 5α reductase activity. A lower activity in PCOS was found for aromatase after adjusting for age and BMI. A higher 11β-hydroxysteroid dehydrogenase (11β-HSD) type 2 and a lower 11β-HSD type 1 activity was found for PCOS for some calculated ratios, whereas other ratios indicated no difference. The activities of 20α- and 20β-hydroxysteroid dehydrogenases (20α/β-HSD) were both lower in PCOS, while 3α-hydroxysteroid dehydrogenase (3α-HSD) activity was higher in PCOS. These results are also depicted in Fig 1.

**Table 2:** Steroid hormone enzyme activities represented by selected steroid hormone metabolite ratios in women with polycystic ovary syndrome compared to healthy women. The available number of participants (N) and median and 25^th^-75^th^ quantile are indicated. Between-group differences are determined by Mann–Whitney U test (MWU). Univariable and multivariable models are calculated by linear regression with transformed steroid hormone metabolite as dependent variable. Univariable models contain the PCOS group as predictor variable (with controls as reference group). Multivariable models contain in addition the covariables age and BMI. The β coefficients and the corresponding 95% confidence intervals (CI) are reported on the transformed scale. Note that only significant different ratios are shown here, while the results for all calculated steroid hormones ratios are displayed in S3 Table.

Enzyme activities and corresponding ratios	Controls			PCOS			MWU	Univariable Models			Multivariable Models		
	N	Median	25^th^-75^th^	N	Median	25^th^-75^th^	*P*	β	95% CI	*P*	β	95% CI	*P*
**21-Hydroxylase**								** **	** **	** **	** **	** **	** **
PTO/THE^a^	64	0.005	0.004–0.008	41	0.003	0.003–0.007	0.0045	-0.368	-0.656;-0.08	0.013	-0.378	-0.689;-0.068	0.017
**3β-hydroxysteroid dehydrogenase**								** **	** **	** **	** **	** **	** **
5PT/THE^b^	64	0.060	0.031–0.097	41	0.087	0.046–0.158	0.025	0.056	0.005;0.107	0.031	0.020	-0.032;0.072	0.45
**11β-hydroxylase**								** **	** **	** **	** **	** **	** **
THS/THE^a^	64	0.023	0.018–0.031	41	0.015	0.011–0.019	<0.001	-0.426	-0.622;-0.23	<0.001	-0.352	-0.559;-0.145	0.0011
**CYP17 global (17α-hydroxylase and 17,20-lyase)**								** **	** **	** **	** **	** **	** **
PD/(AT+ET)^a^	51	0.147	0.073–0.384	41	0.056	0.038–0.069	<0.001	-1.17	-1.56;-0.777	<0.001	-0.943	-1.35;-0.535	<0.001
**17α-hydroxylase global**								** **	** **	** **	** **	** **	** **
THA+THB+5αTHB/THE^b^	64	0.221	0.176–0.279	41	0.157	0.11–0.211	<0.001	-0.068	-0.1;-0.036	<0.001	-0.062	-0.097;-0.027	<0.001
**17α-hydroxylase Δ4-pathway**								** **	** **	** **	** **	** **	** **
PD/17HP^a^	62	4.77	2.88–7.84	41	2.42	1.43–4.1	<0.001	-0.635	-0.927;-0.343	<0.001	-0.540	-0.854;-0.227	<0.001
**17,20-lyase global**								** **	** **	** **	** **	** **	** **
(AT+ET)/THE^b^	52	1.60	1.1–2.17	41	1.48	0.999–2.68	0.77	0.032	-0.119;0.183	0.67	-0.045	-0.2;0.111	0.57
**17,20-lyase Δ5-pathway**								** **	** **	** **	** **	** **	** **
5PT/(DHEA+16OHDHEA)^a^	66	0.230	0.146–0.57	41	0.234	0.12–0.394	0.32	-0.084	-0.45;0.282	0.65	-0.164	-0.56;0.231	0.41
**17,20-lyase Δ4-pathway**								** **	** **	** **	** **	** **	** **
17HP/(AT+ET)^a^	52	0.030	0.02–0.066	41	0.023	0.013–0.032	0.0038	-0.538	-0.864;-0.212	0.0015	-0.423	-0.772;-0.074	0.018
**CYP17 global Δ4- vs. Δ5-pathway**								** **	** **	** **	** **	** **	** **
11βOHAT/(DHEA+16OHDHEA+Δ5diol)^a^	66	1.14	0.554–1.95	41	0.464	0.304–1.26	0.0017	-0.669	-1.04;-0.295	<0.001	-0.470	-0.863;-0.077	0.020
**17β-hydroxysteroid dehydrogenase**								** **	** **	** **	** **	** **	** **
(ET+AT)/(THE+THF+5αTHF)^a^	48	0.834	0.624–1.24	41	0.893	0.563–1.46	0.40	0.087	-0.156;0.33	0.48	-0.050	-0.296;0.196	0.69
**5α-reductase**								** **	** **	** **	** **	** **	** **
ET/AT^a^	53	1.09	0.899–1.36	41	0.798	0.561–1.15	0.0035	-0.282	-0.463;-0.101	0.0026	-0.114	-0.288;0.06	0.20
**Aromatase (CYP19A1)**								** **	** **	** **	** **	** **	** **
testosterone/17β-estradiol^a^	63	2.8	1.64–7.56	33	8.21	3.63–15.7	0.0012	0.725	0.271;1.18	0.0020	0.565	0.087;1.04	0.021
**11β-hydrosteroid dehydrogenase type 2**								** **	** **	** **	** **	** **	** **
(F+E)/(THF+5αTHF+THE)^c^	58	0.812	0.757–0.858	41	0.837	0.797–0.882	0.099	0.053	-0.009;0.115	0.092	0.085	0.018;0.151	0.013
**11β-hydrosteroid dehydrogenase type 1**								** **	** **	** **	** **	** **	** **
THE/(THF+5αTHF)^a^	58	1.08	0.946–1.41	41	1.47	1.19–1.82	<0.001	0.278	0.151;0.404	<0.001	0.272	0.133;0.41	<0.001
**20α-hydrosteroid dehydrogenase**								** **	** **	** **	** **	** **	** **
(THF+5αTHF+THE)/(αC+αCl)^a^	57	1.66	1.28–1.93	41	1.85	1.5–2.32	0.011	0.240	0.089;0.392	0.0022	0.362	0.21;0.515	<0.001
**20β-hydrosteroid dehydrogenase**								** **	** **	** **	** **	** **	** **
(THF+5αTHF+THE)/βC+βCl^a^	59	2.56	1.97–3.26	41	3.04	2.54–4.19	0.0065	0.230	0.08;0.38	0.0030	0.332	0.177;0.486	<0.001
**20α-hydrosteroid dehydrogenase vs. 20β-hydrosteroid dehydrogenase**								** **	** **	** **	** **	** **	** **
(αC+αCl)/(βC+βCl)^a^	61	1.64	1.37–2.07	41	1.54	1.34–2.05	0.85	-0.004	-0.126;0.119	0.95	-0.033	-0.165;0.1	0.62
**3α-hydroxysteroid dehydrogenase**								** **	** **	** **	** **	** **	** **
20αDHF/(THF+5αTHF)^a^	59	0.025	0.017–0.04	40	0.020	0.01–0.032	0.042	-0.365	-0.652;-0.077	0.014	-0.391	-0.701;-0.081	0.014

Abbreviations used for steroid compounds: 17HP: 17-OH-pregnanolone, PT: Pregnanetriol, 5PT: Pregnenetriol, PTO: Pregnanetriolone, PD: Pregnanediol, DHEA: Dehydroepiandrosterone, 16OHDHEA: 16α-OH-dehydroepiandrosterone, Δ5-diol: Androstenediol, 5α3αdiol: Androstanediol, AT: Androsterone, 11βOHAT: 11β-OH-androsterone, ET: Etiocholanolone, THA: 11-dehydro-TH-corticosterone, THB: TH-corticosterone, 5αTHB: Allo-TH-corticosterone, THS: TH-11-deoxycortisol, F: Cortisol, 20αDHF: 20α-DH-cortisol, THF: TH-cortisol, αC: α-Cortol, βC: β-Cortol, 11βOHET: 11β-OH-etiocholanolone, 5αTHF: Allo-TH-cortisol, E: Cortisone, THE: TH-cortisone, αCl: α-Cortolone, βCl: β-Cortolone.

^a^Dependent variable natural log transformed in the models.

^b^Dependent variable square root transformed in the models.

^c^Dependent variable quartic (x^4^) transformed in the models.

### Predicting PCOS by steroid metabolome

**Fig 2:**
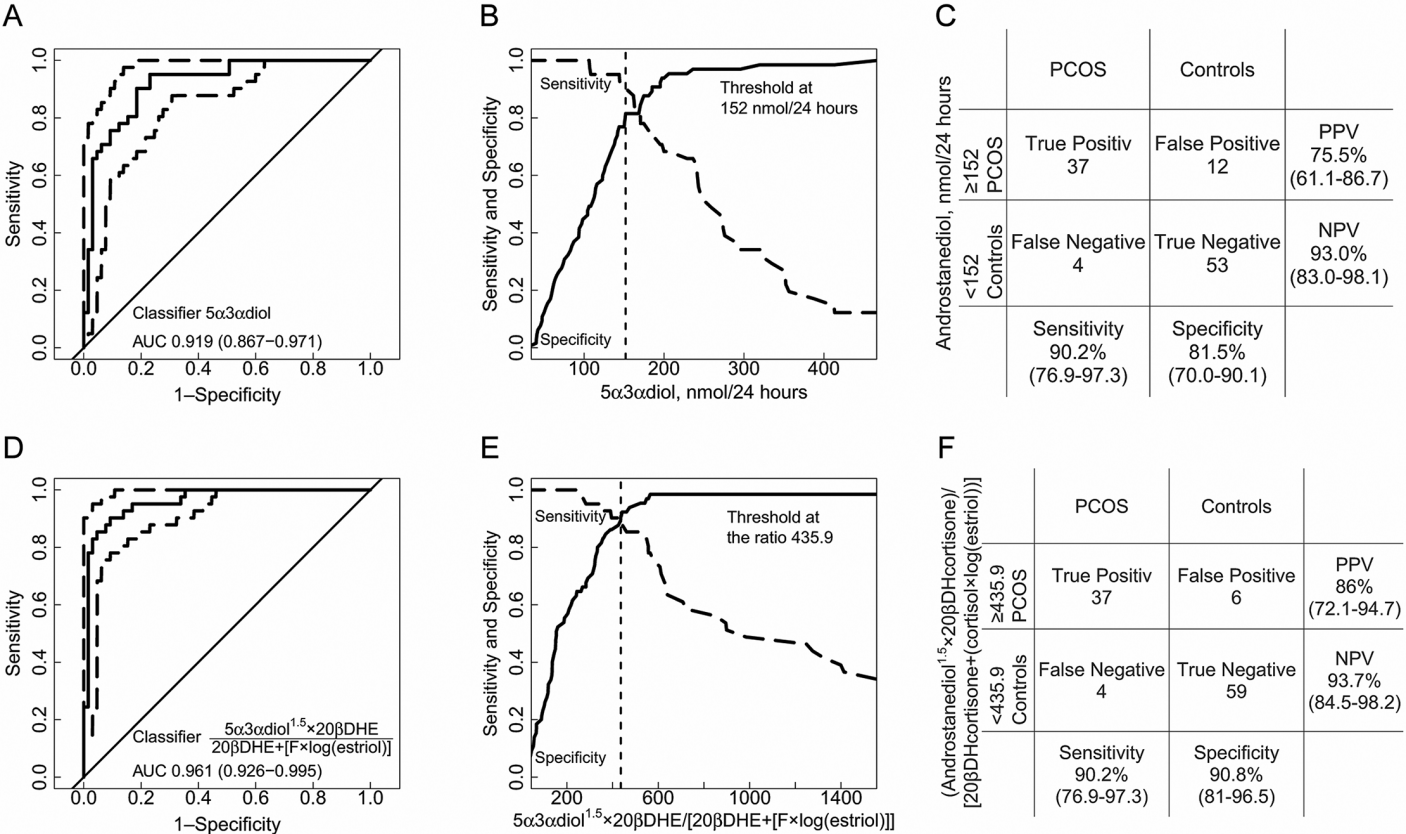
Diagnostic performance of urinary steroid metabolites in the prediction of PCOS. ROC curves for different classifiers of urinary steroid metabolites are shown on the left side, the corresponding plots of sensitivity-specificity versus the classifier are shown in the middle, and corresponding contingency tables on the right hand side. Dashed lines around the ROC curves indicate the 95% CI of the sensitivity at the given specificity. The AUC and its 95% CI is indicated. The dashed vertical lines in the sensitivity-specificity versus classifier plots indicate the threshold where sensitivity and specificity are simultaneously maximized. The main diagnostic performance parameters corresponding to this threshold are indicated. **A-C.** Classifier androstanediol. **D-F.** Classifier: (androstanediol^1.5^×20β-DH-cortisone)/(20β-DH-cortisone+[cortisol× log(estriol)]) represents the best combination of 4 steroid metabolites. Abbreviations: 5α3αdiol: androstanediol, F: cortisol, 20βDHE: 20β-DH-cortisone, PPV: positive predictive value, NPV: negative predictive value, log: natural logarithm.

The diagnostic performance of urinary steroid metabolites in the prediction of PCOS was assessed. Considering each urinary steroid metabolite separately, the androgen androstanediol (5α3αdiol) was the best classifier with the highest AUC in the ROC analysis (0.919, 95% CI: 0.867–0.971; Fig 2A). Maximizing the sensitivity and specificity simultaneously in a sensitivity-specificity-plot (Fig 2B) yielded an optimal threshold for urinary androstanediol at ≥152 nmol/24 hours for the prediction of PCOS with a sensitivity of 90.2 (95% CI: 76.9–97.3), a specificity of 81.5 (95% CI: 70.0–90.1), a positive predictive value of 75.5 (95% CI: 61.9–92.3), and a negative predictive value of 93.0 (95% CI: 82.6–96.5) (Fig 2C).

Performance of urine steroid metabolite ratios for predicting PCOS by systematic calculations was also assessed. The best ratio combining 2 steroid metabolites comprised androstanediol and estriol, and was 5α3αdiol/log(5α3αdiol×estriol) with an AUC of 0.935 (95% CI: 0.889–0.981) under the ROC curve (S1D–S1F Fig). The best combination of 3 urinary steroids was (5α3αdiol×20βDHE)/(20βDHE+cortisol) with an AUC of 0.949 (95% CI: 0.910–0.989) under the ROC curve (S1G–S1I Fig). Finally, the best predictive combination of 4 urinary steroids was (androstanediol^1.5^×20βDHcortisone)/ [20βDHcortisone+(cortisol×log(estriol))] with an AUC of 0.961 (95% CI: 0.926–0.995) under the ROC curve (Fig 2D–2F) yielding a positive predictive value of 86.0% and a negative predictive value of 93.7% for the diagnosis of PCOS at the threshold indicated.

To explore if age and BMI influence these predictors, a multivariable analysis was performed. BMI showed a positive association with all predictors in both PCOS and healthy women (S4 Table and S2 Fig), indicating that body weight increases the tests’ sensitivity while decreasing specificity. For only two predictors age had a different effect on PCOS and healthy controls (S2C and S2D Fig). While in PCOS no age-effect was observed, healthy controls showed decreasing ratios with increasing age suggesting that both tests’ sensitivity and specificity are improving with age.

**Fig 3:**
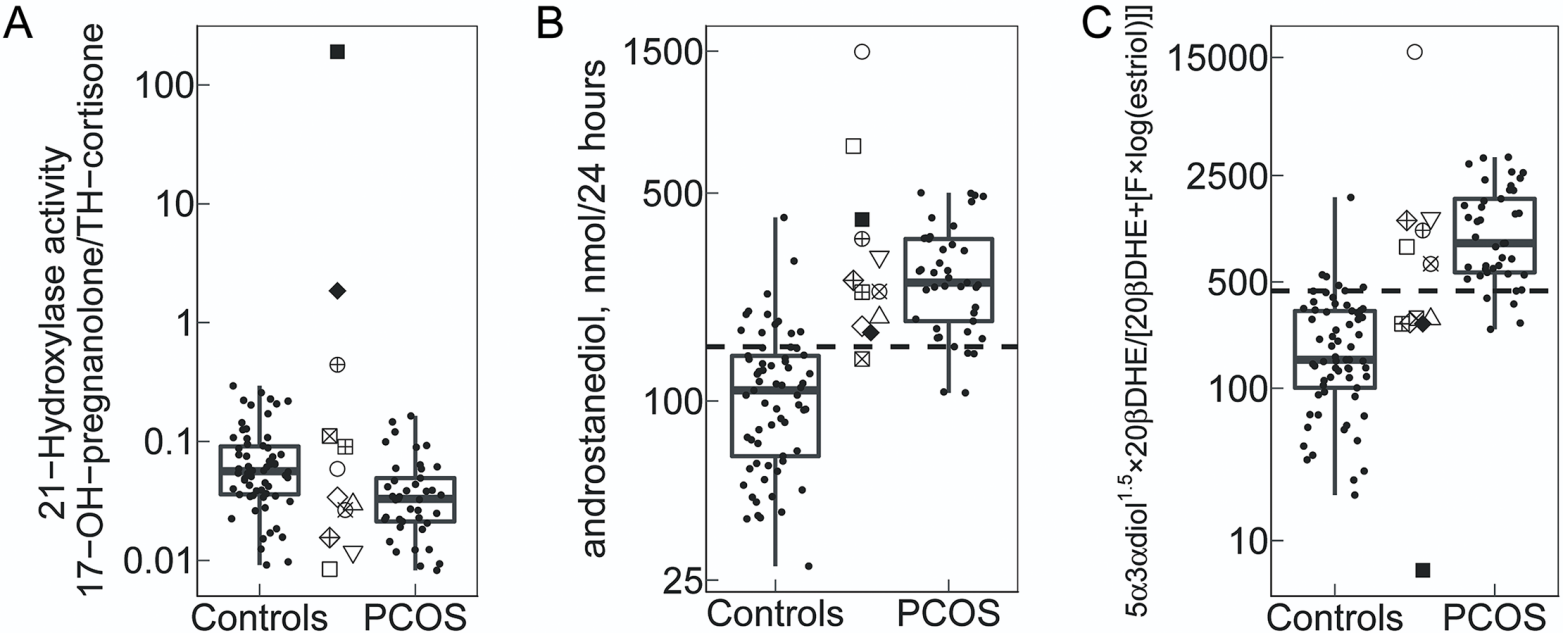
Prospective evaluation of PCOS classifiers. Black points scattered within the boxplots represent study participants. The ten not-filled point symbols between the boxplots represent urine profiles from suspected PCOS women, which were sent to our lab for excluding hyperandrogenism due to 21-hydroxylase deficiency, while the two black-filled point symbols represent urine profiles from women with genetically confirmed 21-hydroxylase deficiency. The dashed horizontal lines indicate the diagnostic thresholds of PCOS classifiers. **A.** 21-hydroxylase activity. A higher ratio of 17-OH-pregnanolone/TH-cortisone indicates a lower 21-hydroxylase activity. **B.** Classifier androstanediol. C. Classifier (androstanediol1.5×20β-DH-cortisone)/(20β-DH-cortisone+[cortisol× log(estriol)]).

Finally for proof of principal, we tested the identified diagnostic classifiers on 12 urinary steroid profiles that were recently analyzed in our GC-MS laboratory: 10 urines were from suspected PCOS women and sent for excluding 21-hydroxylase deficiency, 2 samples originated from subjects with 21-hydroxylase deficiency. Results were compared to study controls and PCOS, and are shown in Fig 3. The 2 samples from subjects with genetically confirmed CYP21A2 mutations showed an increased ratio for 17-OH-prenalonone/TH-cortisone confirming CYP21A2 deficiency (Fig 3A). Among the other 10 samples, 9 samples classified for PCOS according to androstanediol levels (Fig 3B), while 6 of 10 samples qualified for PCOS according to the more complex best ratio calculation including four metabolites (Fig 3C).

## Discussion

Our pilot study suggests that it is possible to specifically diagnose PCOS from urine metabolomics, and not only exclude hyperandrogenic variants of non-classic CAH. Therefore, it appears that PCOS may no longer be diagnosed by exclusion only. Androgen excess is the most characteristic finding in women with PCOS [2]. Nevertheless, no diagnostic test for PCOS based on androgens had been identified so far, although enormous efforts have been undertaken. Reasons for this shortcoming are manifold and include the following: PCOS is a complex disorder likely representing the phenotypical endpoint of multiple underlying disorders leading to androgen excess through several pathways [1,2]. Studies measuring androgens in PCOS lack standardization with respect to preanalytical as well as analytical items and are therefore not comparable. Although in most clinical studies androgens are measured in blood, timing of sampling and specific androgens measured differ. In addition, methods of measurements vary and most immunoassays perform poorly with respect to specificity, availability of normative data as well as standardization across laboratories. Therefore, the scientific community has recommended chromatographic, mass spectrometric techniques for steroid and androgen measurements specifically.

We measured 40 steroid metabolites in 24h-urine specimens from PCOS women and compared them to controls using an established *in-house* GC-MS method [20]. In the past 5–10 years several studies have measured androgens in serum of PCOS women using either GC-MS or LC-MSMS techniques [14,17,23,24], but we found only one recent study assessing the steroids from urine samples [25]. Not surprisingly, all studies (ours included) found elevated androgens of all kinds in PCOS. However, there was no common pattern, and no study suggested a diagnostic marker or formula for discriminating PCOS from healthy controls. Nevertheless, for certain androgens (e.g. total T/DHT [23] or T and androstenedione [24]) a predictive value was reported regarding adverse metabolic outcome in PCOS. Notably, the most recent studies describe involvement of alternative routes for androgen excess in PCOS. Saito et al [14] report a role of the alternative backdoor pathway for androgen overproduction in PCOS. O’Reilly et al [17] found increased 11-oxygenated androgens in PCOS. We found 14/40 urinary steroid metabolites increased in PCOS, among them 9 androgens and 4 glucocorticoids (Fig 1). Highest increase was found for DHEA, the precursor androgen for both adrenal and ovarian androgen production indicating a pathomechanism that targets both organs and/or overall steroidogenesis. Increased androgen metabolites in PCOS were not only comprised in the classic pathway, but also in the alternative backdoor pathway (e.g. androsterone, androstanediol), and they were products of 11-oxygenated androgens (e.g. 11β-hydroxy-androsterone). Thus our data confirm a role of the alternative backdoor pathway and of 11-oxygenated androgens in PCOS. As these pathways of androgen production have been neglected in clinical assessment of PCOS so far, future studies including these metabolites may help in better describing the androgen profile of PCOS and using it as a diagnostic tool. In accordance with that, our calculations revealed androstanediol (a metabolite of the backdoor pathway) as the best single metabolite predictor to discriminate PCOS from controls.

PCOS is defined as not being overlapping with androgen excess due to CAH, mostly 21-hydroxylase deficiency [1,2]. Some studies of ovarian steroidogenesis suggest that in PCOS activities of HSD3B2 and CYP17-17,20 lyase are enhanced [26]. However, studies looking at steroid enzyme activities assessed by calculating steroid conversion ratios reveal ambiguous results. Increased 5α reductase activity in PCOS has been suggested from clinical studies [15,25], and was also suggested from immunohistochemical studies looking at ovarian tissues [13]. In our study, we found an increase in 21-hydroxylase, 11β-hydroxylase, 17α-hydroxylase/17,20 lyase (Δ4) as well as 3α-HSD activity in PCOS (Fig 1). By contrast, we found no clear difference for 3β-HSD activity. Overall, these findings in PCOS do not fit a pattern for a specific steroid biosynthesis disorder, but they indicate overall enhanced steroidogenesis and towards androgens specifically. Thus PCOS clearly separates from CAH.

Similar to the urine steroid profiling study by Blumenfeld [25], we found increased androgen and glucocorticoid metabolites in PCOS. In both studies, 5α reductase activity seemed increased when looking at its activity within the backdoor pathway (11βOHET/11βOHAT), but not with respect to its activity in the degradation of mineralocorticoids (THB/5αTHB) and glucocorticoids (THF/5αTHF). However, this effect seemed associated with BMI in both studies. As 5α reductase activity is essential to yield androgen precursor metabolites for DHT production, this indicates that in PCOS an increase in BMI will enhance 5α-dependent androgen production. In line with that, clinical studies unambiguously show an improvement of hyperandrogenism in PCOS women with weight loss [2].

Concerning 11β-hydroxysteroid dehydrogenase activities, we found an increased type 2 and decreased type 1 activity, but no change in absolute cortisol excretion. Blumenfeld suggested a decrease in type 1 activity from one calculated ratio [25]. Diminished HSD11B1 activity has been previously reported in PCOS [27,28,29,30] and may result in a shift of steroidogenesis towards the more active glucocorticoid products associated with hypercortisolemic adverse effects often manifesting as the metabolic syndrome. Finally, other studies found an increase in 20α-HSD activity (lower ratio of THF+αTHF+THE/αC+αCl) [25,28], while our study revealed diminished 20αHSD and 20β-HSD activities, but an increase in 3α-HSD activity assessed by the conversion of αTHF and THF to 20αDHF. 20α-HSD activity is mainly promoted by AKR1C1, but may also be promoted by any other member of the AKR1C superfamily of aldo/keto reductases, which are also known as 3αHSDs. Generally, 3αHSDs enzymes are expressed tissue specific and are important for the metabolism of glucocorticoids, progesterones, prostaglandins, and bile acid precursors [7]. Concerning steroidogenesis, 3αHSD activity is highly promoted by AKR1C4 and AKR1C2. In the gonads and the adrenals 3αHSD catalyzes the conversion of 5α-androstanedione to androsterone and from 17α-OH-dihydro-progesterone to 17α-OH-allopregnanolone in the backdoor pathway [9]. Similarly, it catalyzes the conversion of highly active DHT to almost inactive androstanediol in the prostate. In previous studies, we have shown that mutations in AKR1C2/4 cause 46,XY undermasculinization [10], and that in ovarian tissues from PCOS women expression of AKR1C2/4 seemed enhanced [13]. Thus increased 3αHSD activity might be characteristic for hyperandrogenic PCOS similar to increased 17α-hydroxylase/17,20 lyase activity and 5α reductase activity. Excess activity of all these enzymes in concert might explain why androstanediol accumulates with PCOS.

Our search for a diagnostic marker from urine steroid profiling using AUC/ROC curve analysis yielded androstanediol as the best single metabolite for classifying PCOS against controls. This metabolite is comprised in the backdoor steroid path and may be easily converted to the most active steroid DHT by oxidative 3αHSD, which is likely promoted by RODH in steroid organs [7]. In fact, RODH expression was found rather increased in PCOS ovarian tissues [13]. Taken together a role of the backdoor pathway for excess androgen production in PCOS seems likely.

To predict PCOS, the best combination including up to four steroids was a ratio comprising androstanediol, estriol, 20βDHcortisone and cortisol. This ratio was significantly increased in PCOS compared to controls at a threshold value of ≥435. Taking ratios for steroid analysis bears the advantage that they are less influenced by different laboratory methods than quantitative steroid excretion values. Thus, such diagnostic ratios should allow comparison of data between laboratories as has been shown for normative values of steroid enzyme activities [22]. Applying these diagnostic tools to some preliminary data sets of suspected PCOS women available from our lab, we found that PCOS diagnosis could be supported in 9/10 subjects using androstanediol as single classifier and in 6/10 subjects using the best ratio comprising of 4 steroid metabolites. Importantly, two steroid profiles originating from suboptimal treated patients with 21-hydroxylase CAH clearly discriminated from both controls and PCOS when looking at the 21-hydroxylase activity and at the newly developed PCOS activity ratios. In comparison to the classifier androstanediol, the use of the ratio comprising of 4 steroid metabolites reduces the number of false positives for PCOS.

Limitations of our study are the relative small sample number and the relatively poor clinical characterization of the PCOS subjects. However, compared to the study of Blumenfeld [25], in which only 13 samples were studied, we studied 41 PCOS samples and 66 controls. Clinical characterization of PCOS subjects is rather difficult as the phenotypical spectrum is broad. Thus finding a biochemical classifier that discriminates PCOS from non-PCOS is of great clinical interest. Of course, better metabolic characterization of PCOS samples in future studies may allow to correlate the steroid data with adverse metabolic outcome, which impacts treatment decisions. Another disadvantage of our study for practicability is maybe that we performed steroid profiling from 24h-urine samples and not spot urines or serum. However, it should be feasible to test within short time, whether the identified PCOS classifiers may also be used on timed spot urines or serum samples.

In conclusion, our urinary steroid profiling study reveals androstanediol, estriol, 20βDHcortisone, and cortisol as promising diagnostic markers for PCOS. These so far unsuspected steroids in the diagnostic workup of PCOS were identified using novel, unbiased approaches for data analysis. Future studies will aim at confirming their diagnostic use in spot urine and serum specimen as well as testing their predictive value for adverse metabolic outcome.

## Supporting information

S1 Fig Diagnostic performance of urinary steroid metabolites in the prediction of PCOS. (DOC)

S2 Fig Association between PCOS, age and body mass index (BMI) with four classifiers derived from the urine steroid hormone metabolome for the prediction of PCOS. (DOC)

S1 Table Comparison of baseline characteristics. (DOC)

S2 Table Comparison of steroid hormone metabolite excretion and its association with PCOS. (DOC)

S3 Table Comparison of steroid hormone metabolite ratios to assess steroid enzyme activities. (DOC)

S4 Table Association between PCOS, age and body mass index (BMI) with four classifiers derived from the urine steroid hormone metabolome to predict PCOS. (DOC)

## References

[1] Azziz R, Carmina E, Dewailly D, Diamanti-Kandarakis E, Escobar-Morreale HF, Futterweit W, et al (2009) The Androgen Excess and PCOS Society criteria for the polycystic ovary syndrome: the complete task force report. Fertil Steril 91: 456–488. 10.1016/j.fertnstert.2008.06.035 18950759

[2] Legro RS, Arslanian SA, Ehrmann DA, Hoeger KM, Murad MH, Pasquali R, et al (2013) Diagnosis and treatment of polycystic ovary syndrome: an Endocrine Society clinical practice guideline. J Clin Endocrinol Metab 98: 4565–4592. 10.1210/jc.2013-2350 24151290 PMC5399492

[3] Peigne M, Dewailly D (2014) Long term complications of polycystic ovary syndrome (PCOS). Annales d'endocrinologie 75: 194–199. 10.1016/j.ando.2014.07.111 25156132

[4] Gibson-Helm M, Teede H, Dunaif A, Dokras A (2017) Delayed Diagnosis and a Lack of Information Associated With Dissatisfaction in Women With Polycystic Ovary Syndrome. The Journal of clinical endocrinology and metabolism 102: 604–612. 10.1210/jc.2016-2963 27906550 PMC6283441

[5] Bardin CW, Lipsett MB (1967) Testosterone and androstenedione blood production rates in normal women and women with idiopathic hirsutism or polycystic ovaries. J Clin Invest 46: 891–902. 10.1172/JCI105588 6025489 PMC297090

[6] Piltonen T, Koivunen R, Morin-Papunen L, Ruokonen A, Huhtaniemi IT, Tapanainen JS. (2002) Ovarian and adrenal steroid production: regulatory role of LH/HCG. Hum Reprod 17: 620–624. 11870113 10.1093/humrep/17.3.620

[7] Miller WL, Auchus RJ (2011) The molecular biology, biochemistry, and physiology of human steroidogenesis and its disorders. Endocr Rev 32: 81–151. 10.1210/er.2010-0013 21051590 PMC3365799

[8] Auchus RJ (2004) The backdoor pathway to dihydrotestosterone. Trends Endocrinol Metab 15: 432–438. 10.1016/j.tem.2004.09.004 15519890

[9] Biason-Lauber A, Miller WL, Pandey AV, Fluck CE (2013) Of marsupials and men: "Backdoor" dihydrotestosterone synthesis in male sexual differentiation. Mol Cell Endocrinol 371: 124–132. 10.1016/j.mce.2013.01.017 23376007

[10] Fluck CE, Meyer-Boni M, Pandey AV, Kempna P, Miller WL, Schoenle EJ, et al (2011) Why boys will be boys: two pathways of fetal testicular androgen biosynthesis are needed for male sexual differentiation. Am J Hum Genet 89: 201–218. 10.1016/j.ajhg.2011.06.009 21802064 PMC3155178

[11] Homma K, Hasegawa T, Nagai T, Adachi M, Horikawa R, Fujiwara I, et al (2006) Urine steroid hormone profile analysis in cytochrome P450 oxidoreductase deficiency: implication for the backdoor pathway to dihydrotestosterone. J Clin Endocrinol Metab 91: 2643–2649. 10.1210/jc.2005-2460 16608896

[12] Kamrath C, Hochberg Z, Hartmann MF, Remer T, Wudy SA (2012) Increased activation of the alternative "backdoor" pathway in patients with 21-hydroxylase deficiency: evidence from urinary steroid hormone analysis. J Clin Endocrinol Metab 97: E367–375. 10.1210/jc.2011-1997 22170725

[13] Marti N, Galvan JA, Pandey AV, Trippel M, Tapia C, Muller M, et al (2017) Genes and proteins of the alternative steroid backdoor pathway for dihydrotestosterone synthesis are expressed in the human ovary and seem enhanced in the polycystic ovary syndrome. Mol Cell Endocrinol 441: 116–123. 10.1016/j.mce.2016.07.029 27471004

[14] Saito K, Matsuzaki T, Iwasa T, Miyado M, Saito H, Hasegawa T, et al (2016) Steroidogenic pathways involved in androgen biosynthesis in eumenorrheic women and patients with polycystic ovary syndrome. J Steroid Biochem Mol Biol 158: 31–37. 10.1016/j.jsbmb.2016.02.010 26877255

[15] Fassnacht M, Schlenz N, Schneider SB, Wudy SA, Allolio B, Arlt W. (2003) Beyond adrenal and ovarian androgen generation: Increased peripheral 5 alpha-reductase activity in women with polycystic ovary syndrome. J Clin Endocrinol Metab 88: 2760–2766. 10.1210/jc.2002-021875 12788885

[16] Turcu A, Smith JM, Auchus R, Rainey WE (2014) Adrenal androgens and androgen precursors-definition, synthesis, regulation and physiologic actions. Compr Physiol 4: 1369–1381. 10.1002/cphy.c140006 25428847 PMC4437668

[17] O'Reilly MW, Kempegowda P, Jenkinson C, Taylor AE, Quanson JL, Storbeck KH, et al (2017) 11-Oxygenated C19 Steroids Are the Predominant Androgens in Polycystic Ovary Syndrome. J Clin Endocrinol Metab 102: 840–848. 10.1210/jc.2016-3285 27901631 PMC5460696

[18] Pruijm M, Ponte B, Ackermann D, Vuistiner P, Paccaud F, Guessous I, et al (2013) Heritability, determinants and reference values of renal length: a family-based population study. Eur Radiol. 10.1007/s00330-013-2900-4 23712436

[19] Ponte B, Pruijm M, Ackermann D, Vuistiner P, Eisenberger U, Guessous I, et al (2014) Reference values and factors associated with renal resistive index in a family-based population study—ONLINE SUPPLEMENT. Hypertension 63: 136–142. 10.1161/HYPERTENSIONAHA.113.02321 24126174

[20] Dhayat NA, Frey AC, Frey BM, d'Uscio CH, Vogt B, Rousson V, et al (2015) Estimation of reference curves for the urinary steroid metabolome in the first year of life in healthy children: tracing the complexity of human postnatal steroidogenesis. J Steroid Biochem Mol Biol. 10.1016/j.jsbmb.2015.07.024 26297192

[21] Matthews DR, Hosker JP, Rudenski AS, Naylor BA, Treacher DF, Turner RC. (1985) Homeostasis model assessment: insulin resistance and beta-cell function from fasting plasma glucose and insulin concentrations in man. Diabetologia 28: 412–419. 3899825 10.1007/BF00280883

[22] Dhayat NA, Dick B, Frey BM, d'Uscio CH, Vogt B, Fluck CE. (2017) Androgen biosynthesis during minipuberty favors the backdoor pathway over the classic pathway: Insights into enzyme activities and steroid fluxes in healthy infants during the first year of life from the urinary steroid metabolome. J Steroid Biochem Mol Biol 165: 312–322. 10.1016/j.jsbmb.2016.07.009 27471148

[23] Munzker J, Hofer D, Trummer C, Ulbing M, Harger A, Pieber T, et al (2015) Testosterone to dihydrotestosterone ratio as a new biomarker for an adverse metabolic phenotype in the polycystic ovary syndrome. J Clin Endocrinol Metab 100: 653–660. 10.1210/jc.2014-2523 25387259

[24] O'Reilly MW, Taylor AE, Crabtree NJ, Hughes BA, Capper F, Crowley RK, et al (2014) Hyperandrogenemia predicts metabolic phenotype in polycystic ovary syndrome: the utility of serum androstenedione. J Clin Endocrinol Metab 99: 1027–1036. 10.1210/jc.2013-3399 24423344 PMC3955250

[25] Blumenfeld Z, Kaidar G, Zuckerman-Levin N, Dumin E, Knopf C, Hochberg Z. (2016) Cortisol-Metabolizing Enzymes in Polycystic Ovary Syndrome. Clin Med Insights Reprod Health 10: 9–13. 10.4137/CMRH.S35567 27168731 PMC4859446

[26] Nelson VL, Qin KN, Rosenfield RL, Wood JR, Penning TM, Legro RS, et al (2001) The biochemical basis for increased testosterone production in theca cells propagated from patients with polycystic ovary syndrome. J Clin Endocrinol Metab 86: 5925–5933. 10.1210/jcem.86.12.8088 11739466

[27] Gambineri A, Vicennati V, Genghini S, Tomassoni F, Pagotto U, Pasquali R, et al (2006) Genetic variation in 11beta-hydroxysteroid dehydrogenase type 1 predicts adrenal hyperandrogenism among lean women with polycystic ovary syndrome. J Clin Endocrinol Metab 91: 2295–2302. 10.1210/jc.2005-2222 16551740

[28] Tsilchorozidou T, Honour JW, Conway GS (2003) Altered cortisol metabolism in polycystic ovary syndrome: insulin enhances 5alpha-reduction but not the elevated adrenal steroid production rates. J Clin Endocrinol Metab 88: 5907–5913. 10.1210/jc.2003-030240 14671189

[29] Rodin A, Thakkar H, Taylor N, Clayton R (1994) Hyperandrogenism in polycystic ovary syndrome. Evidence of dysregulation of 11 beta-hydroxysteroid dehydrogenase. N Engl J Med 330: 460–465. 10.1056/NEJM199402173300703 8289851

[30] Walker BR, Rodin A, Taylor NF, Clayton RN (2000) Endogenous inhibitors of 11beta-hydroxysteroid dehydrogenase type 1 do not explain abnormal cortisol metabolism in polycystic ovary syndrome. Clin Endocrinol (Oxf) 52: 77–80. 10651756 10.1046/j.1365-2265.2000.00893.x

