# Supplementary material for: Urinary steroid profiling in women hints at a diagnostic signature of the polycystic ovary syndrome: A pilot study considering neglected steroid metabolites
Source: PLoS One. 2018 Oct 11;13(10):e0203903. doi: 10.1371/journal.pone.0203903 (PMC6181287; doi:10.1371/journal.pone.0203903)
Supplement: S1 Fig — (DOC) [file pone.0203903.s001.doc]

**Supporting information**

**

**

**S1 Figure. Diagnostic performance of urinary steroid metabolites in the prediction of PCOS.** ROC curves for different classifiers of urinary steroid metabolites are shown on the left side, the corresponding plots of sensitivity-specificity versus the classifier are shown in the middle, and corresponding contingency tables on the right side. Dashed lines around the ROC curves indicate the 95% CI of the sensitivity at the given specificity. The AUC and its 95% CI is indicated. The dashed vertical lines in the sensitivity-specificity versus classifier plots indicate the threshold where sensitivity and specificity are simultaneously maximized. The main diagnostic performance parameters corresponding to this threshold are indicated. **A-C.** Classifier androstanediol. **D-F.** The classifier androstanediol/log(androstanediol×estriol) represents the best combination of 2 steroid metabolites found to achieve the highest possible AUC under the ROC curve. **G-I.** The classifier (androstanediol×20β-DH-cortisone)/(20β-DH-cortisone+cortisol) represents the best combination of 3 steroid metabolites found. **J-L.** Classifier (androstanediol^1.5^×20β-DH-cortisone)/(20β-DH-cortisone+ [cortisol×log(estriol)] represents the best combination of 4 steroid metabolites found. Abbreviations: 5α3αdiol: androstanediol, F: cortisol, 20βDHE: 20β-DH-cortisone, PPV: positive predictive value, NPV: negative predictive value, log: natural logarithm.
