# Supplementary material for: Urinary steroid profiling in women hints at a diagnostic signature of the polycystic ovary syndrome: A pilot study considering neglected steroid metabolites
Source: PLoS One. 2018 Oct 11;13(10):e0203903. doi: 10.1371/journal.pone.0203903 (PMC6181287; doi:10.1371/journal.pone.0203903)
Supplement: S2 Fig — (DOC) [file pone.0203903.s002.doc]

**Supporting information**

**

**

**S2 Figure. Association between PCOS, age and body mass index (BMI) with four classifiers derived from the urine steroid hormone metabolome for the prediction of PCOS.** All models were calculated by linear regression and contain PCOS women and Controls and the covariables age and BMI. Natural logarithm transformation was applied to all four classifiers as dependent variables on y-axis. The model coefficients are indicated in Supplemental Table 2. Figures on the left side (**A, C, E, G**) visualize the association between age and the log transformed classifier separately for PCOS women and control women adjusted for BMI. Figures on the right side (**B, D, F, H**) visualize the association between BMI and the log transformed classifier separately for PCOS and controls adjusted for age. Figures with the same classifier (dependent variable) derives from the same model, thus, a total of four multivariable models are described here. Solid black lines indicate regression lines and the shaped grey area the 95% confidence interval, respectively.
