# Supplementary material for: Urinary steroid profiling in women hints at a diagnostic signature of the polycystic ovary syndrome: A pilot study considering neglected steroid metabolites
Source: PLoS One. 2018 Oct 11;13(10):e0203903. doi: 10.1371/journal.pone.0203903 (PMC6181287; doi:10.1371/journal.pone.0203903)
Supplement: S1 Table — (DOC) [file pone.0203903.s003.doc]

**Supporting information**

| **S1 Table. Comparison of baseline characteristics.** The available number of participants (N) for the PCOS and control group and median and 25^th^-75^th^ quantile are indicated. Between-group differences are determined by Mann–Whitney U test (MWU). |
| --- |

| **Characteristics** |  | **Controls** | | |  | **PCOS** | | |  | **MWU** |
| --- | --- | --- | --- | --- | --- | --- | --- | --- | --- | --- |
|  |  | **N** | **Median** | **25^th^-75^th^** |  | **N** | **Median** | **25^th^-75^th^** |  | ***P*** |
| Age, years |  | 66 | 34 | 28-42 |  | 41 | 27 | 24-32 |  | <0.001 |
| Weight, kg |  | 66 | 63 | 55-70 |  | 41 | 68 | 58-71 |  | 0.26 |
| Height, cm |  | 66 | 168 | 162-171 |  | 41 | 164 | 158-171 |  | 0.071 |
| Body mass index, kg/m^2^ |  | 66 | 22.4 | 19.5-25.7 |  | 41 | 24.0 | 21-28 |  | 0.065 |
| Systolique blood pressure, mmHg |  | 66 | 109 | 102-116 |  | 37 | 115 | 110-125 |  | 0.0019 |
| Diastolique blood pressure, mmHg |  | 66 | 71 | 68-78 |  | 37 | 72 | 62-80 |  | 0.84 |
| Glucose fasting, plasma, mmol/L |  | 66 | 4.765 | 4.4-5.1 |  | 17 | 4.7 | 4.5-5.3 |  | 0.39 |
| Insulin, mU/L |  | 63 | 3.2 | 1-5.8 |  | 14 | 16.6 | 13.7-22.3 |  | <0.001 |
| HOMA-IR |  | 63 | 0.65 | 0.24-1.12 |  | 13 | 3.74 | 2.77-4.58 |  | <0.001 |
| HOMA-β |  | 63 | 58.2 | 20.8-113 |  | 13 | 284 | 106-357 |  | <0.001 |
| Urine volume 24h, mL |  | 66 | 1775 | 1283-2017 |  | 41 | 1670 | 1250-2200 |  | 0.95 |
