# Supplementary material for: Urinary steroid profiling in women hints at a diagnostic signature of the polycystic ovary syndrome: A pilot study considering neglected steroid metabolites
Source: PLoS One. 2018 Oct 11;13(10):e0203903. doi: 10.1371/journal.pone.0203903 (PMC6181287; doi:10.1371/journal.pone.0203903)
Supplement: S2 Table — (DOC) [file pone.0203903.s004.doc]

**Supporting information**

**S2 Table. Comparison of steroid hormone metabolite excretion and its association with PCOS.** The available number of participants (N) and median and 25^th^-75^th^ quantile are indicated. Between-group differences are determined by Mann–Whitney U test (MWU). Univariable and multivariable models are calculated by linear regression with transformed steroid hormone metabolite as dependent variable. Univariable models contain the PCOS group as predictor variable (with controls as reference group). Multivariable models contain in addition the covariables age and BMI. The β coefficients and the corresponding 95% confidence intervals (CI) are reported on the transformed scale.

| **Steroid hormone, nmol/24h** | **Controls** | | |  | **PCOS** | | |  | **MWU** | **Univariable Models** | | |  | **Multivariable Models** | | |
| --- | --- | --- | --- | --- | --- | --- | --- | --- | --- | --- | --- | --- | --- | --- | --- | --- |
|  | **N** | **Median** | **25^th^-75^th^** |  | **N** | **Median** | **25^th^-75^th^** |  | ***P*** | **β** | **95% CI** | ***P*** |  | **β** | **95% CI** | ***P*** |
| 17-OH-pregnanolone^a^ | 64 | 264 | 179-532 |  | 41 | 326 | 188-433 |  | 0.80 | -0.076 | -0.399;0.247 | 0.64 |  | -0.151 | -0.498;0.195 | 0.39 |
| Pregnanetriol^b^ | 62 | 1452 | 1135-2209 |  | 41 | 1892 | 1214-2636 |  | 0.064 | 4.22 | -0.175;8.61 | 0.060 |  | 1.75 | -2.79;6.29 | 0.45 |
| Pregnenetriol^b^ | 66 | 280 | 155-542 |  | 41 | 792 | 544-1364 |  | <0.001 | 10.7 | 6.37;15.1 | <0.001 |  | 7.72 | 3.31;12.1 | <0.001 |
| Pregnanetriolone^a^ | 66 | 27 | 18-43 |  | 41 | 32 | 20-46 |  | 0.49 | 0.115 | -0.195;0.424 | 0.46 |  | 0.021 | -0.312;0.353 | 0.90 |
| Pregnanediol^a^ | 64 | 1063 | 673-2885 |  | 41 | 652 | 405-1121 |  | 0.0019 | -0.675 | -1.04;-0.311 | <0.001 |  | -0.669 | -1.07;-0.271 | 0.0012 |
| DHEA^a^ | 66 | 293 | 136-853 |  | 41 | 1435 | 390-3895 |  | <0.001 | 1.27 | 0.712;1.83 | <0.001 |  | 1.03 | 0.437;1.62 | <0.001 |
| 16α-OH-DHEA^a^ | 66 | 676 | 314-1213 |  | 41 | 1577 | 701-3321 |  | <0.001 | 0.740 | 0.289;1.19 | 0.0015 |  | 0.740 | 0.289;1.19 | 0.0015 |
| Androstenediol^a^ | 66 | 205 | 125-430 |  | 41 | 622 | 405-1314 |  | <0.001 | 1.07 | 0.705;1.43 | <0.001 |  | 0.858 | 0.483;1.23 | <0.001 |
| Testosterone^a^ | 63 | 34 | 21-58 |  | 33 | 52 | 34-84 |  | 0.013 | 0.449 | 0.106;0.793 | 0.011 |  | 0.427 | 0.05;0.804 | 0.027 |
| 5α-DH-testosterone^a^ | 65 | 36 | 23-55 |  | 33 | 56 | 44-88 |  | 0.0057 | 0.477 | 0.148;0.805 | 0.0049 |  | 0.387 | 0.029;0.746 | 0.035 |
| Androstanediol/5α3αdiol^a^ | 65 | 108 | 65-142 |  | 41 | 250 | 185-350 |  | <0.001 | 0.930 | 0.735;1.13 | <0.001 |  | 0.886 | 0.68;1.09 | <0.001 |
| Androsterone^b^ | 57 | 3983 | 2651-5433 |  | 41 | 8354 | 4909-11808 |  | <0.001 | 24.9 | 15.7;34 | <0.001 |  | 14.7 | 6.31;23 | <0.001 |
| 5α-androstenetriol^a^ | 66 | 803 | 579-1186 |  | 41 | 1068 | 774-1490 |  | 0.044 | 0.173 | -0.132;0.479 | 0.26 |  | -0.083 | -0.388;0.222 | 0.59 |
| 11β-OH-androsterone^b^ | 66 | 1385 | 1049-2048 |  | 41 | 2210 | 1618-3263 |  | <0.001 | 9.80 | 5.35;14.2 | <0.001 |  | 8.73 | 4.2;13.3 | <0.001 |
| Etiocholanolone^b^ | 61 | 4075 | 2823-5709 |  | 41 | 5893 | 4558-8210 |  | <0.001 | 13.5 | 6.3;20.6 | <0.001 |  | 9.65 | 2.13;17.2 | 0.012 |
| 17β-estradiol^a^ | 66 | 10 | 6-16 |  | 33 | 7 | 5-12 |  | 0.060 | -0.278 | -0.591;0.035 | 0.081 |  | -0.138 | -0.453;0.178 | 0.39 |
| Estriol^a^ | 66 | 29 | 16-49 |  | 41 | 21 | 8-34 |  | 0.027 | -0.444 | -0.809;-0.079 | 0.018 |  | -0.491 | -0.877;-0.105 | 0.013 |
| 11-deoxy-TH-corticosterone^a^ | 66 | 26 | 15-43 |  | 41 | 25 | 16-32 |  | 0.55 | -0.165 | -0.476;0.146 | 0.30 |  | -0.158 | -0.494;0.178 | 0.35 |
| 11-dehydro-TH-corticosterone^b^ | 66 | 230 | 146-353 |  | 41 | 299 | 226-384 |  | 0.037 | 2.05 | 0.238;3.85 | 0.027 |  | 1.92 | -0.041;3.88 | 0.055 |
| 18-OH-11-dehydro-TH-corticosterone^a^ | 60 | 83 | 57-136 |  | 40 | 112 | 81-186 |  | 0.051 | 0.266 | -0.078;0.611 | 0.13 |  | 0.214 | -0.156;0.584 | 0.25 |
| TH-corticosterone^b^ | 66 | 294 | 217-436 |  | 41 | 359 | 220-476 |  | 0.24 | 1.16 | -0.81;3.13 | 0.25 |  | 1.63 | -0.456;3.72 | 0.12 |
| allo-TH-corticosterone^b^ | 66 | 586 | 415-840 |  | 41 | 675 | 459-1103 |  | 0.18 | 2.38 | -0.674;5.44 | 0.13 |  | 1.49 | -1.74;4.72 | 0.36 |
| TH-aldosterone^a^ | 66 | 64 | 36-95 |  | 41 | 58 | 25-80 |  | 0.38 | -0.111 | -0.423;0.201 | 0.48 |  | -0.090 | -0.429;0.249 | 0.60 |
| TH-11-deoxycortisol^b^ | 66 | 122 | 98-161 |  | 41 | 121 | 90-190 |  | 0.52 | 0.446 | -0.598;1.49 | 0.40 |  | 0.495 | -0.616;1.6 | 0.38 |
| Cortisol^b^ | 66 | 227 | 156-325 |  | 41 | 174 | 128-287 |  | 0.13 | -1.26 | -2.83;0.319 | 0.12 |  | -0.983 | -2.69;0.722 | 0.26 |
| 6β-OH-cortisol^a^ | 66 | 222 | 147-348 |  | 41 | 319 | 189-445 |  | 0.025 | 0.238 | -0.012;0.489 | 0.062 |  | 0.256 | -0.016;0.529 | 0.065 |
| 18-OH-cortisol^b^ | 61 | 434 | 301-607 |  | 39 | 676 | 448-924 |  | <0.001 | 5.75 | 3.1;8.39 | <0.001 |  | 5.89 | 3.02;8.76 | <0.001 |
| 20α-DH-cortisol^a^ | 66 | 125 | 84-164 |  | 40 | 112 | 67-199 |  | 0.75 | -0.043 | -0.276;0.19 | 0.71 |  | -0.099 | -0.354;0.156 | 0.44 |
| TH-cortisol^b^ | 59 | 2770 | 1926-3439 |  | 41 | 3613 | 2603-4404 |  | 0.0017 | 8.06 | 2.85;13.3 | 0.0028 |  | 7.91 | 2.72;13.1 | 0.0032 |
| α-Cortol^b^ | 66 | 565 | 423-720 |  | 41 | 584 | 470-798 |  | 0.59 | 0.364 | -1.83;2.56 | 0.74 |  | -1.02 | -3.02;0.974 | 0.31 |
| β-Cortol^b^ | 65 | 669 | 503-953 |  | 41 | 783 | 570-1220 |  | 0.47 | 0.609 | -2.55;3.77 | 0.70 |  | -1.02 | -4.16;2.12 | 0.52 |
| 11β-OH-etiocholanolone^b^ | 66 | 872 | 410-1196 |  | 40 | 1037 | 255-1640 |  | 0.51 | 1.87 | -2.92;6.67 | 0.44 |  | 5.75 | 0.837;10.7 | 0.022 |
| Allo-TH-cortisol^b^ | 63 | 1761 | 1102-2717 |  | 41 | 2502 | 1507-3069 |  | 0.018 | 7.81 | 1.38;14.2 | 0.018 |  | 4.55 | -1.88;11 | 0.16 |
| Cortisone^b^ | 66 | 384 | 278-510 |  | 41 | 411 | 309-516 |  | 0.56 | 0.431 | -1.37;2.23 | 0.64 |  | 0.384 | -1.57;2.34 | 0.70 |
| 20α-DH-cortisone^a^ | 66 | 49 | 38-65 |  | 41 | 42 | 30-67 |  | 0.24 | -0.089 | -0.31;0.132 | 0.43 |  | -0.175 | -0.41;0.06 | 0.14 |
| 20β-DH-cortisone^b^ | 66 | 132 | 106-175 |  | 41 | 173 | 120-270 |  | 0.015 | 1.57 | 0.361;2.78 | 0.011 |  | 0.899 | -0.353;2.15 | 0.16 |
| TH-cortisone^b^ | 64 | 5551 | 3394-7209 |  | 41 | 8559 | 5651-13063 |  | <0.001 | 23.8 | 14.8;32.9 | <0.001 |  | 21.2 | 12;30.4 | <0.001 |
| α-Cortolone^b^ | 64 | 2413 | 1750-2940 |  | 41 | 2645 | 2136-3124 |  | 0.20 | 2.45 | -1.87;6.76 | 0.26 |  | -1.78 | -5.5;1.94 | 0.35 |
| β-Cortolone^b^ | 64 | 985 | 720-1321 |  | 41 | 1085 | 879-1471 |  | 0.24 | 1.68 | -1.52;4.88 | 0.30 |  | -0.737 | -3.83;2.35 | 0.64 |
| 11-keto-etiocholanolone^b^ | 66 | 893 | 465-1253 |  | 41 | 836 | 438-1357 |  | 0.87 | -0.097 | -4.11;3.92 | 0.96 |  | 1.47 | -2.82;5.76 | 0.50 |
| ^a^The dependent variable was natural log transformed in the models.  ^b^The dependent variable was square root transformed in the models. | | | | | | | | | | | | | | | | |
