# Supplementary material for: Urinary steroid profiling in women hints at a diagnostic signature of the polycystic ovary syndrome: A pilot study considering neglected steroid metabolites
Source: PLoS One. 2018 Oct 11;13(10):e0203903. doi: 10.1371/journal.pone.0203903 (PMC6181287; doi:10.1371/journal.pone.0203903)
Supplement: S3 Table — (DOC) [file pone.0203903.s005.doc]

**Supporting information**

**S3 Table.** **Comparison of steroid hormone metabolite ratios to assess steroid enzyme activities.** The available number of participants (N) and median and 25^th^-75^th^ quantile are indicated. Between-group differences are determined by Mann–Whitney U test (MWU). Univariable and multivariable models are calculated by linear regression with transformed steroid hormone metabolite as dependent variable. Univariable models contain the PCOS group as predictor variable (with controls as reference group). Multivariable models contain in addition the covariables age and BMI. The β coefficients and the corresponding 95% confidence intervals (CI) are reported on the transformed scale.

|  | | | | | | | | | | | | | | | | |
| --- | --- | --- | --- | --- | --- | --- | --- | --- | --- | --- | --- | --- | --- | --- | --- | --- |
| **Enzyme activities and corresponding ratios** | **Controls** | | |  | **PCOS** | | |  | **MWU** | **Univariable Models** | | |  | **Multivariable Models** | | |
|  | **N** | **Median** | **25^th^-75^th^** |  | **N** | **Median** | **25^th^-75^th^** |  | ***P*** | **β** | **95% CI** | ***P*** |  | **β** | **95% CI** | ***P*** |
| **21-Hydroxylase** | | | | | | | | | |  |  |  |  |  |  |  |
| PTO/THE^a^ | 64 | 0.005 | 0.004-0.008 |  | 41 | 0.003 | 0.003-0.007 |  | 0.0045 | -0.368 | -0.656;-0.08 | 0.013 |  | -0.378 | -0.689;-0.068 | 0.017 |
| PTO/(THE+THF+5αTHF)^a^ | 58 | 0.003 | 0.002-0.004 |  | 41 | 0.002 | 0.001-0.004 |  | 0.041 | -0.253 | -0.554;0.048 | 0.100 |  | -0.267 | -0.596;0.061 | 0.11 |
| 17HP/THE^a^ | 62 | 0.056 | 0.036-0.091 |  | 41 | 0.033 | 0.021-0.049 |  | <0.001 | -0.558 | -0.87;-0.246 | <0.001 |  | -0.565 | -0.906;-0.223 | 0.0014 |
| 17HP/(THE+THF+5αTHF)^a^ | 58 | 0.029 | 0.02-0.042 |  | 41 | 0.019 | 0.012-0.029 |  | 0.0054 | -0.442 | -0.765;-0.118 | 0.0079 |  | -0.453 | -0.807;-0.098 | 0.013 |
| PT/THE^a^ | 61 | 0.287 | 0.211-0.387 |  | 41 | 0.210 | 0.153-0.3 |  | 0.0047 | -0.333 | -0.533;-0.133 | 0.0013 |  | -0.385 | -0.597;-0.173 | <0.001 |
| PT/(THE+THF+5αTHF)^a^ | 57 | 0.155 | 0.106-0.207 |  | 41 | 0.119 | 0.094-0.175 |  | 0.069 | -0.207 | -0.414;-0.001 | 0.049 |  | -0.268 | -0.487;-0.049 | 0.017 |
| (PTO+17HP+PT)/THE^a^ | 59 | 0.355 | 0.271-0.511 |  | 41 | 0.250 | 0.175-0.346 |  | 0.0024 | -0.365 | -0.575;-0.156 | <0.001 |  | -0.419 | -0.644;-0.194 | <0.001 |
| (PTO+17HP+PT)/(THE+THF+5αTHF)^a^ | 57 | 0.195 | 0.136-0.239 |  | 41 | 0.152 | 0.112-0.205 |  | 0.033 | -0.242 | -0.455;-0.028 | 0.027 |  | -0.295 | -0.523;-0.066 | 0.012 |
| **3β-hydroxysteroid dehydrogenase** | | | | | | | | | |  |  |  |  |  |  |  |
| 5PT/THE^b^ | 64 | 0.060 | 0.031-0.097 |  | 41 | 0.087 | 0.046-0.158 |  | 0.025 | 0.056 | 0.005;0.107 | 0.031 |  | 0.020 | -0.032;0.072 | 0.45 |
| 5PT/(THE+THF+5αTHF)^b^ | 58 | 0.027 | 0.016-0.05 |  | 41 | 0.052 | 0.028-0.092 |  | 0.0026 | 0.057 | 0.018;0.096 | 0.0046 |  | 0.029 | -0.012;0.07 | 0.16 |
| DHEA/THE^a^ | 64 | 0.052 | 0.028-0.169 |  | 41 | 0.152 | 0.05-0.576 |  | 0.0045 | 0.781 | 0.257;1.31 | 0.0039 |  | 0.638 | 0.071;1.2 | 0.028 |
| DHEA/(THE+THF+5αTHF)^a^ | 58 | 0.028 | 0.015-0.096 |  | 41 | 0.094 | 0.026-0.331 |  | 0.0027 | 0.861 | 0.32;1.4 | 0.0021 |  | 0.685 | 0.094;1.28 | 0.024 |
| DHEA+16OHDHEA/THE^a^ | 64 | 0.214 | 0.092-0.436 |  | 41 | 0.362 | 0.135-0.909 |  | 0.020 | 0.524 | 0.091;0.958 | 0.018 |  | 0.323 | -0.137;0.783 | 0.17 |
| DHEA+16OHDHEA/(THE+THF+5αTHF)^a^ | 58 | 0.120 | 0.05-0.262 |  | 41 | 0.243 | 0.087-0.56 |  | 0.012 | 0.611 | 0.155;1.07 | 0.009 |  | 0.375 | -0.113;0.863 | 0.13 |
| **11β-hydroxylase** | | | | | | | | | |  |  |  |  |  |  |  |
| THS/THE^a^ | 64 | 0.023 | 0.018-0.031 |  | 41 | 0.015 | 0.011-0.019 |  | <0.001 | -0.426 | -0.622;-0.23 | <0.001 |  | -0.352 | -0.559;-0.145 | 0.0011 |
| THS/(THE+THF+5αTHF)^a^ | 58 | 0.012 | 0.009-0.017 |  | 41 | 0.008 | 0.007-0.01 |  | <0.001 | -0.314 | -0.51;-0.117 | 0.0020 |  | -0.239 | -0.447;-0.031 | 0.025 |
| **CYP17 global (17α-hydroxylase and 17,20-lyase)** | | | | | | | | | |  |  |  |  |  |  |  |
| PD/(AT+ET)^a^ | 51 | 0.147 | 0.073-0.384 |  | 41 | 0.056 | 0.038-0.069 |  | <0.001 | -1.17 | -1.56;-0.777 | <0.001 |  | -0.943 | -1.35;-0.535 | <0.001 |
| (THA+THB+5αTHB)/(AT+ET)^a^ | 53 | 0.150 | 0.096-0.213 |  | 41 | 0.087 | 0.07-0.165 |  | 0.017 | -0.301 | -0.557;-0.045 | 0.022 |  | -0.145 | -0.405;0.115 | 0.27 |
| **17α-hydroxylase global** | | | | | | | | | |  |  |  |  |  |  |  |
| THA+THB+5αTHB/THE^b^ | 64 | 0.221 | 0.176-0.279 |  | 41 | 0.157 | 0.11-0.211 |  | <0.001 | -0.068 | -0.1;-0.036 | <0.001 |  | -0.062 | -0.097;-0.027 | <0.001 |
| THA+THB+5αTHB/(THE+THF+5αTHF)^b^ | 58 | 0.120 | 0.086-0.138 |  | 41 | 0.095 | 0.069-0.121 |  | 0.0071 | -0.028 | -0.051;-0.006 | 0.014 |  | -0.023 | -0.047;0.001 | 0.063 |
| **17α-hydroxylase Δ4-pathway** | | | | | | | | | |  |  |  |  |  |  |  |
| PD/17HP^a^ | 62 | 4.77 | 2.88-7.84 |  | 41 | 2.42 | 1.43-4.1 |  | <0.001 | -0.635 | -0.927;-0.343 | <0.001 |  | -0.540 | -0.854;-0.227 | <0.001 |
| PD/PT^a^ | 60 | 0.807 | 0.512-1.59 |  | 41 | 0.442 | 0.262-0.528 |  | <0.001 | -0.861 | -1.16;-0.562 | <0.001 |  | -0.700 | -1.01;-0.388 | <0.001 |
| PD/(PT+17HP)^a^ | 58 | 0.681 | 0.439-1.43 |  | 41 | 0.346 | 0.226-0.49 |  | <0.001 | -0.834 | -1.12;-0.543 | <0.001 |  | -0.679 | -0.983;-0.375 | <0.001 |
| **17,20-lyase global** | | | | | | | | | |  |  |  |  |  |  |  |
| (AT+ET)/THE^b^ | 52 | 1.60 | 1.1-2.17 |  | 41 | 1.48 | 0.999-2.68 |  | 0.77 | 0.032 | -0.119;0.183 | 0.67 |  | -0.045 | -0.2;0.111 | 0.57 |
| (AT+ET)/(THE+THF+5αTHF)^b^ | 48 | 0.834 | 0.624-1.24 |  | 41 | 0.893 | 0.563-1.46 |  | 0.40 | 0.055 | -0.057;0.168 | 0.33 |  | -0.006 | -0.121;0.109 | 0.92 |
| **17,20-lyase Δ5-pathway** | | | | | | | | | |  |  |  |  |  |  |  |
| 5PT/(DHEA+16OHDHEA)^a^ | 66 | 0.230 | 0.146-0.57 |  | 41 | 0.234 | 0.12-0.394 |  | 0.32 | -0.084 | -0.45;0.282 | 0.65 |  | -0.164 | -0.56;0.231 | 0.41 |
| 5PT/(Δ5diol)^b^ | 66 | 1.36 | 0.789-2.32 |  | 41 | 1.05 | 0.756-1.5 |  | 0.17 | -0.110 | -0.272;0.052 | 0.18 |  | -0.183 | -0.355;-0.01 | 0.038 |
| 5PT/(DHEA+16OHDHEA+Δ5-diol)^a^ | 66 | 0.189 | 0.119-0.421 |  | 41 | 0.159 | 0.106-0.321 |  | 0.22 | -0.117 | -0.467;0.233 | 0.51 |  | -0.218 | -0.595;0.158 | 0.25 |
| **17,20-lyase Δ4-pathway** | | | | | | | | | |  |  |  |  |  |  |  |
| 17HP/11βOHAT^a^ | 64 | 0.192 | 0.119-0.357 |  | 41 | 0.134 | 0.08-0.192 |  | 0.012 | -0.449 | -0.765;-0.134 | 0.0057 |  | -0.462 | -0.808;-0.117 | 0.0093 |
| PT/11βOHAT^a^ | 62 | 1.07 | 0.693-1.49 |  | 41 | 0.828 | 0.589-1.33 |  | 0.067 | -0.211 | -0.43;0.007 | 0.058 |  | -0.272 | -0.503;-0.041 | 0.022 |
| (17HP+PT)11βOHAT^a^ | 60 | 1.26 | 0.811-1.83 |  | 41 | 0.931 | 0.708-1.49 |  | 0.069 | -0.235 | -0.464;-0.005 | 0.045 |  | -0.292 | -0.539;-0.045 | 0.021 |
| 17HP/(AT+ET)^a^ | 52 | 0.030 | 0.02-0.066 |  | 41 | 0.023 | 0.013-0.032 |  | 0.0038 | -0.538 | -0.864;-0.212 | 0.0015 |  | -0.423 | -0.772;-0.074 | 0.018 |
| PT/(AT+ET)^a^ | 51 | 0.180 | 0.123-0.251 |  | 41 | 0.136 | 0.095-0.181 |  | 0.0062 | -0.299 | -0.503;-0.095 | 0.0045 |  | -0.236 | -0.454;-0.019 | 0.033 |
| (17HP+PT)/(AT+ET)^a^ | 50 | 0.214 | 0.14-0.337 |  | 41 | 0.167 | 0.119-0.204 |  | 0.0070 | -0.335 | -0.555;-0.115 | 0.0032 |  | -0.261 | -0.496;-0.027 | 0.029 |

| **S3 Table continued** |  |  |  |  |  |  |  |  |  |  |  |  |  |  |  |  |
| --- | --- | --- | --- | --- | --- | --- | --- | --- | --- | --- | --- | --- | --- | --- | --- | --- |
| **CYP17 global Δ4- vs. Δ5-pathway** | | | | | | | | | |  |  |  |  |  |  |  |
| 11βOHAT/(DHEA+16OHDHEA)^a^ | 66 | 1.37 | 0.696-2.47 |  | 41 | 0.570 | 0.353-1.57 |  | 0.0044 | -0.635 | -1.04;-0.232 | 0.0023 |  | -0.416 | -0.84;0.008 | 0.054 |
| 11βOHAT/Δ5diol^b^ | 66 | 7.03 | 4.43-10.3 |  | 41 | 3.60 | 2.3-5.16 |  | <0.001 | -0.788 | -1.13;-0.449 | <0.001 |  | -0.623 | -0.98;-0.265 | <0.001 |
| 11βOHAT/(DHEA+16OHDHEA+Δ5diol)^a^ | 66 | 1.14 | 0.554-1.95 |  | 41 | 0.464 | 0.304-1.26 |  | 0.0017 | -0.669 | -1.04;-0.295 | <0.001 |  | -0.470 | -0.863;-0.077 | 0.020 |
| **P450 oxidoreductase** | | | | | | | | | |  |  |  |  |  |  |  |
| (17HP+PT)/THE^a^ | 59 | 0.345 | 0.264-0.501 |  | 41 | 0.247 | 0.172-0.344 |  | 0.0030 | -0.367 | -0.58;-0.154 | <0.001 |  | -0.420 | -0.649;-0.191 | <0.001 |
| (17HP+PT)/(THE+THF+5αTHF)^a^ | 57 | 0.190 | 0.132-0.236 |  | 41 | 0.145 | 0.106-0.201 |  | 0.036 | -0.243 | -0.46;-0.027 | 0.028 |  | -0.296 | -0.528;-0.063 | 0.013 |
| PD/THE^a^ | 63 | 0.198 | 0.127-0.479 |  | 41 | 0.078 | 0.046-0.138 |  | <0.001 | -1.19 | -1.58;-0.798 | <0.001 |  | -1.08 | -1.49;-0.669 | <0.001 |
| PD/(THE+THF+5αTHF)^a^ | 57 | 0.107 | 0.064-0.276 |  | 41 | 0.045 | 0.027-0.088 |  | <0.001 | -1.08 | -1.5;-0.664 | <0.001 |  | -0.975 | -1.42;-0.528 | <0.001 |
| **17β-hydroxysteroid dehydrogenase** | | | | | | | | | |  |  |  |  |  |  |  |
| (ET+AT)/(THE+THF+5αTHF)^a^ | 48 | 0.834 | 0.624-1.24 |  | 41 | 0.893 | 0.563-1.46 |  | 0.40 | 0.087 | -0.156;0.33 | 0.48 |  | -0.050 | -0.296;0.196 | 0.69 |
| **Alternative androgen backdoor pathway after the 17,20 lyase vs. classic pathway** | | | | | | | | | |  |  |  |  |  |  |  |
| AT/ET^a^ | 53 | 0.918 | 0.733-1.112 |  | 41 | 1.253 | 0.872-1.783 |  | 0.0035 | 0.282 | 0.101;0.463 | 0.0026 |  | 0.114 | -0.06;0.288 | 0.20 |
| **5α-reductase** | | | | | | | | | |  |  |  |  |  |  |  |
| ET/AT^a^ | 53 | 1.09 | 0.899-1.36 |  | 41 | 0.798 | 0.561-1.15 |  | 0.0035 | -0.282 | -0.463;-0.101 | 0.0026 |  | -0.114 | -0.288;0.06 | 0.20 |
| 11βOHET/11βOHAT^b^ | 66 | 0.576 | 0.361-0.886 |  | 40 | 0.431 | 0.14-0.696 |  | 0.023 | -0.115 | -0.229;-0.001 | 0.0480 |  | -0.017 | -0.132; 0.097 | 0.76 |
| THF/5αTHF^a^ | 59 | 1.54 | 1.06-2.04 |  | 41 | 1.43 | 1.01-2 |  | 0.78 | -0.014 | -0.236;0.208 | 0.90 |  | 0.134 | -0.098;0.366 | 0.26 |
| THB/5αTHB^a^ | 66 | 0.534 | 0.383-0.68 |  | 41 | 0.513 | 0.328-0.719 |  | 0.62 | -0.060 | -0.247;0.127 | 0.53 |  | 0.056 | -0.138;0.25 | 0.57 |
| **Aromatase (CYP19A1)** | | | | | | | | | |  |  |  |  |  |  |  |
| testosterone/17β-estradiol^a^ | 63 | 2.8 | 1.64-7.56 |  | 33 | 8.21 | 3.63-15.7 |  | 0.0012 | 0.725 | 0.271;1.18 | 0.0020 |  | 0.565 | 0.087;1.04 | 0.021 |
| **11β-hydrosteroid dehydrogenase type 2** | | | | | | | | | |  |  |  |  |  |  |  |
| F/E^a^ | 66 | 0.618 | 0.452-0.811 |  | 41 | 0.474 | 0.385-0.599 |  | 0.0071 | -0.208 | -0.366;-0.05 | 0.010 |  | -0.171 | -0.342;0.001 | 0.051 |
| (THF+5αTHF)/THE^a^ | 58 | 0.929 | 0.71-1.06 |  | 41 | 0.681 | 0.549-0.839 |  | <0.001 | -0.278 | -0.404;-0.151 | <0.001 |  | -0.272 | -0.41;-0.133 | <0.001 |
| (αC+βC)/(αCl+βCl)^a^ | 61 | 0.372 | 0.319-0.467 |  | 41 | 0.381 | 0.309-0.449 |  | 0.47 | -0.053 | -0.161;0.055 | 0.33 |  | -0.003 | -0.116;0.11 | 0.96 |
| (F+E)/(THF+5αTHF+THE)^a^ | 58 | 0.061 | 0.046-0.081 |  | 41 | 0.038 | 0.027-0.049 |  | <0.001 | -0.467 | -0.654;-0.280 | <0.001 |  | -0.399 | -0.591;-0.207 | <0.001 |
| **11β-hydrosteroid dehydrogenase type 1** | | | | | | | | | |  |  |  |  |  |  |  |
| THE/(THF+5αTHF)^a^ | 58 | 1.08 | 0.946-1.41 |  | 41 | 1.47 | 1.19-1.82 |  | <0.001 | 0.278 | 0.151;0.404 | <0.001 |  | 0.272 | 0.133;0.41 | <0.001 |
| (αCl+βCl)/(αC+βC)^a^ | 61 | 2.69 | 2.14-3.14 |  | 41 | 2.63 | 2.23-3.24 |  | 0.47 | 0.053 | -0.055;0.161 | 0.33 |  | 0.003 | -0.11;0.116 | 0.96 |
| **20α-hydrosteroid dehydrogenase** | | | | | | | | | |  |  |  |  |  |  |  |
| (THF+5αTHF+THE)/(αC+αCl)^a^ | 56 | 3.46 | 2.90-4.07 |  | 41 | 4.74 | 3.62-6.24 |  | <0.001 | 0.388 | 0.238;0.537 | <0.001 |  | 0.512 | 0.361;0.664 | <0.001 |
| **20β-hydrosteroid dehydrogenase** | | | | | | | | | |  |  |  |  |  |  |  |
| (THF+5αTHF+THE)/βC+βCl^a^ | 58 | 5.43 | 4.63-6.81 |  | 41 | 8.55 | 5.98-11.81 |  | <0.001 | 0.378 | 0.232;0.524 | <0.001 |  | 0.478 | 0.325;0.630 | <0.001 |
| **20α-hydrosteroid dehydrogenase vs. 20β-hydrosteroid dehydrogenase** | | | | | | | | | |  |  |  |  |  |  |  |
| (αC+αCl)/(βC+βCl)^a^ | 61 | 1.64 | 1.37-2.07 |  | 41 | 1.54 | 1.34-2.05 |  | 0.85 | -0.004 | -0.126;0.119 | 0.95 |  | -0.033 | -0.165;0.1 | 0.62 |
| **3α-hydroxysteroid dehydrogenase** | | | | | | | | | |  |  |  |  |  |  |  |
| 20αDHF/(THF+5αTHF)^a^ | 59 | 0.025 | 0.017-0.04 |  | 40 | 0.020 | 0.01-0.032 |  | 0.042 | -0.365 | -0.652;-0.077 | 0.014 |  | -0.391 | -0.701;-0.081 | 0.014 |
| Abbreviations used for steroid compounds: 17HP: 17-OH-pregnanolone, PT: Pregnanetriol, 5PT: Pregnenetriol, PTO: Pregnanetriolone, PD: Pregnanediol, DHEA: Dehydroepiandrosterone, 16OHDHEA: 16α-OH-dehydroepiandrosterone, Δ5-diol: Androstenediol, 5α3αdiol: Androstanediol, AT: Androsterone, 11βOHAT: 11β-OH-androsterone, ET: Etiocholanolone, THA: 11-dehydro-TH-corticosterone, THB: TH-corticosterone, 5αTHB: Allo-TH-corticosterone, THS: TH-11-deoxycortisol, F: Cortisol, 20αDHF: 20α-DH-cortisol, THF: TH-cortisol, αC: α-Cortol, βC: β-Cortol, 11βOHET: 11β-OH-etiocholanolone, 5αTHF: Allo-TH-cortisol, E: Cortisone, THE: TH-cortisone, αCl: α-Cortolone, βCl: β-Cortolone. ^a^The dependent variable was natural log transformed in the models. ^b^The dependent variable was square root transformed in the models. | | | | | | | | | | | | | | | | |
