# Supplementary material for: Urinary steroid profiling in women hints at a diagnostic signature of the polycystic ovary syndrome: A pilot study considering neglected steroid metabolites
Source: PLoS One. 2018 Oct 11;13(10):e0203903. doi: 10.1371/journal.pone.0203903 (PMC6181287; doi:10.1371/journal.pone.0203903)
Supplement: S4 Table — (DOC) [file pone.0203903.s006.doc]

**Supporting information**

**S4 Table. Association between PCOS, age and body mass index (BMI) with four classifiers derived from the urine steroid hormone metabolome to predict PCOS.** All four models were calculated by linear regression and are multivariable containing the PCOS and control group and the covariables age and BMI. The presence of interaction between PCOS and age and PCOS and BMI was considered and backward selection was carried out to eliminate interaction terms with a *P* value ≥ 0.10. All main effects were kept in the model irrespective of their significance. Natural logarithm transformation was applied to all four classifiers as dependent variables. The β coefficients and the corresponding 95% confidence intervals (CI) are reported on the transformed scale. *P* values for the predictor variables PCOS, age, BMI and the interaction between PCOS and age are indicated.

|  | **PCOS** | | |  | **Age** | | |  | **BMI** | | |  | **Interaction:PCOS×Age** | | |
| --- | --- | --- | --- | --- | --- | --- | --- | --- | --- | --- | --- | --- | --- | --- | --- |
| **Classifier/Dependent variable** | **β** | **95% CI** | ***P*** |  | **β** | **95% CI** | ***P*** |  | **β** | **95% CI** | ***P*** |  | **β** | **95% CI** | ***P*** |
| androstanediol | 0.886 | 0.68;1.09 | <0.001 |  | -0.0008 | -0.0125;0.0109 | 0.89 |  | 0.0242 | 0.0047;0.0436 | 0.016 |  | - | -;- | - |
| androstanediol/  log(androstanediol×estriol) | 0.839 | 0.655;1.02 | <0.001 |  | -0.0016 | -0.0121;0.0089 | 0.77 |  | 0.0151 | -0.0024;0.0325 | 0.089 |  | - | -;- | - |
| (androstanediol×20β-DH-cortisone)/  (20β-DH-cortisone+cortisol) | 0.052 | -0.762;0.865 | 0.90 |  | -0.021 | -0.0353;-0.0066 | 0.0046 |  | 0.04 | 0.0199;0.0601 | <0.001 |  | 0.0332 | 0.0066;0.0597 | 0.015 |
| (androstanediol^1.5^×20β-DH-cortisone)/  (20β-DH-cortisone+[cortisol×log(estriol)] | 0.251 | -1.01;1.51 | 0.69 |  | -0.031 | -0.0533;-0.0088 | 0.0068 |  | 0.0444 | 0.0132;0.0756 | 0.0057 |  | 0.0478 | 0.0067;0.089 | 0.023 |
